# Paramylon isolated from *Euglena gracilis* EOD-1 extends lifespan through activation of DAF-16-mediated antioxidant pathway via *clec-196* in *Caenorhabditis elegans*

**DOI:** 10.1038/s41598-025-26199-3

**Published:** 2025-11-26

**Authors:** Yasuki Higashimura, Mina Isobe, Kiyoshi Miura, Natsumi Desaka, Hitomi Nishikawa, Norihisa Nishida, Junko Naito, Takanori Kawano, Tomihiro Miyada, Yuji Naito

**Affiliations:** 1 Department of Food Science, Ishikawa Prefectural University, Nonoichi, Ishikawa, 921-8836 Japan; 2 Department of Clinical Nutrition, Kawasaki University of Medical Welfare, Okayama, 701-0193 Japan; 3 Department of Public Health and Environmental Medicine, The Jikei University School of Medicine, Tokyo, 105-8461 Japan; 4 Kobelco Eco-Solutions Co., Ltd, Kobe, 651-2241 Japan; 5 Department of Human Immunology and Nutrition Science, Kyoto Prefectural University of Medicine, Kyoto, 602-8566 Japan

**Keywords:** B-glucan, Clec-196, Dectin-1, Longevity, Nematode, RNA interference, Cell biology, Molecular biology, Physiology

## Abstract

Paramylon (PM), an insoluble β-1,3-glucan produced by *Euglena gracilis*, reportedly possesses immunomodulatory and metabolic regulatory effects. However, its effect on longevity remains unclear. For this study, using *Caenorhabditis elegans* as a model, we evaluated lifespan-extending effects of PM and elucidated its underlying molecular mechanisms. Dietary PM supplementation prolonged the *C. elegans* lifespan significantly without affecting either growth or fertility, indicating the effects as independent of caloric restriction. Findings indicate that PM intake strongly upregulated *clec-196*, an ortholog of the β-glucan receptor DECTIN-1. Also, RNA interference of *clec-196* eliminated PM-induced lifespan extension, indicating *clec-196* as necessary for the physiological response to PM. Moreover, PM supplementation increased expression of *jnk-1* and *daf-16*, which were suppressed when *clec-196* was knocked down, suggesting that *clec-196* functions upstream of the JNK-1/DAF-16 pathway. The lifespan-extending effect of PM was completely absent in loss-of-function mutant of *daf-16*, underscoring its indispensable role. Furthermore, PM feeding activated the DAF-16-mediated antioxidant pathway, as evidenced by upregulation of antioxidant genes and by suppression of hydrogen peroxide accumulation. These findings suggest that PM might serve as a functional food ingredient exhibiting anti-aging potential.

**Supplementary Information:**

The online version contains supplementary material available at 10.1038/s41598-025-26199-3.

## Introduction

Because of changes in diet, environment, and lifestyle, aging populations in modern societies are increasing rapidly. Aging, an inevitable biological process that affects all organs and cells, is recognized as a common risk factor for numerous health-threatening diseases including cancer, cardiovascular diseases, and neurodegenerative disorders^1^. As understanding of the complex process of aging has advanced, twelve hallmarks that define this phenomenon, including genomic instability, mitochondrial dysfunction, and chronic inflammation, have been proposed^2^. Many of these hallmarks are associated with excessive oxidative stress and the persistence of low-grade inflammatory states^3,4^. Indeed, several studies have suggested that certain nutrients and food-derived compounds with antioxidant and/or anti-inflammatory properties may delay the onset of age-related symptoms and thereby contribute to lifespan extension^5,6^.

Dietary fiber is an important component of a healthy diet. Among various types of dietary fiber, β-glucans are distributed widely in nature. They are present even in fungi and algae, but their structural and physicochemical properties are known to vary depending on the source organism^7^. The biological activities of β-glucans have also been shown to depend on these structural and physicochemical characteristics. For instance, β-1,3 − 1,4-glucans, which are predominantly found in cereals, are viscous and fermentable dietary fibers, reportedly playing a crucially important role in the improvement of obesity-related metabolic disorders^8,9^. In contrast, β-1,3 − 1,6-glucans, which are the principal cell wall components of fungi such as yeast and mushrooms, are largely insoluble fibers. They reportedly enhance immune responses through the C-type lectin receptor DECTIN-1^10,11^.

Paramylon (PM), a polysaccharide produced as a characteristic intracellular storage material in *Euglena spp.*, is classified as an insoluble dietary fiber. Structurally, PM consists of linear β-1,3-glucan chains that adopt a triple helical conformation, which aggregate further to form crystalline granules. This unique structure lends high chemical stability to PM, rendering it insoluble in water (even in hot water) and making it resistant to enzymatic degradation by β-1,3-glucanase^7,12^. It is noteworthy that PM has been shown to act as a ligand for DECTIN-1, even in its particulate form^13^. With regard to its physiological functions, both human clinical and animal studies have demonstrated several health benefits of PM, including immunomodulatory effects^14,15^, prevention of obesity-related parameters^16,17^, and alleviation of fatigue^18^. In addition, PM reportedly ameliorates liver dysfunction in rats by attenuating oxidative damage and by enhancing reductase activity in hepatic tissues^19^. The fermentability and effects of PM on the gut microbiota persist as subjects of ongoing debate^20–22^, but the PM structural and chemical stability suggest that its influence on microbial composition is minimal. Therefore, PM might be regarded as a unique dietary fiber with properties that are distinct from those of previously studied fibers.

*Caenorhabditis elegans*, a bacterivorous soil-dwelling nematode, has been used widely as an experiment system for biological studies because of its morphological simplicity, transparent body, ease of cultivation, and amenability to genetic analysis^23^. Moreover, its short lifespan and high experimental reproducibility are advantageous for aging research. Genetic and environmental factors, including dietary components, influence the *C. elegans* lifespan. Genes that regulate lifespan are associated with several evolutionarily conserved signaling pathways involved in aging, such as the insulin/insulin-like growth factor-1 signaling (IIS) pathway^24,25^. In addition, several pattern recognition receptors that respond to extracellular stimuli are conserved in this organism, including Toll-like receptors and several C-type lectin-like domain (CTLD)-containing proteins including DECTIN-1^26^. Consequently, *C. elegans* serves as an excellent model for investigating the effects on longevity exerted by external factors such as nutritional stimuli and food components. Furthermore, most studies using *C. elegans* have employed the canonical Bristol N2 strain, which is typically maintained on a monoxenic diet consisting solely of *Escherichia coli* OP50 (OP50), a standard laboratory food source. Other microorganisms are removed routinely via hypochlorite treatment. Consequently, the N2 strain is devoid of any microbes, including those in the gut and on the body surface^27^. This characteristic makes *C. elegans* a powerful tool for evaluating the effects of nutritional stimuli and food components without the confounding influence of gut microbiota.

Given this context, this study evaluated whether PM can extend the lifespan of *C. elegans*. Furthermore, RNA interference (RNAi) and various mutant strains were used to elucidate the mechanisms underlying PM-mediated lifespan extension.

## Results

### PM extends the *C. elegans* lifespan without participation of caloric restriction

**Fig. 1:**
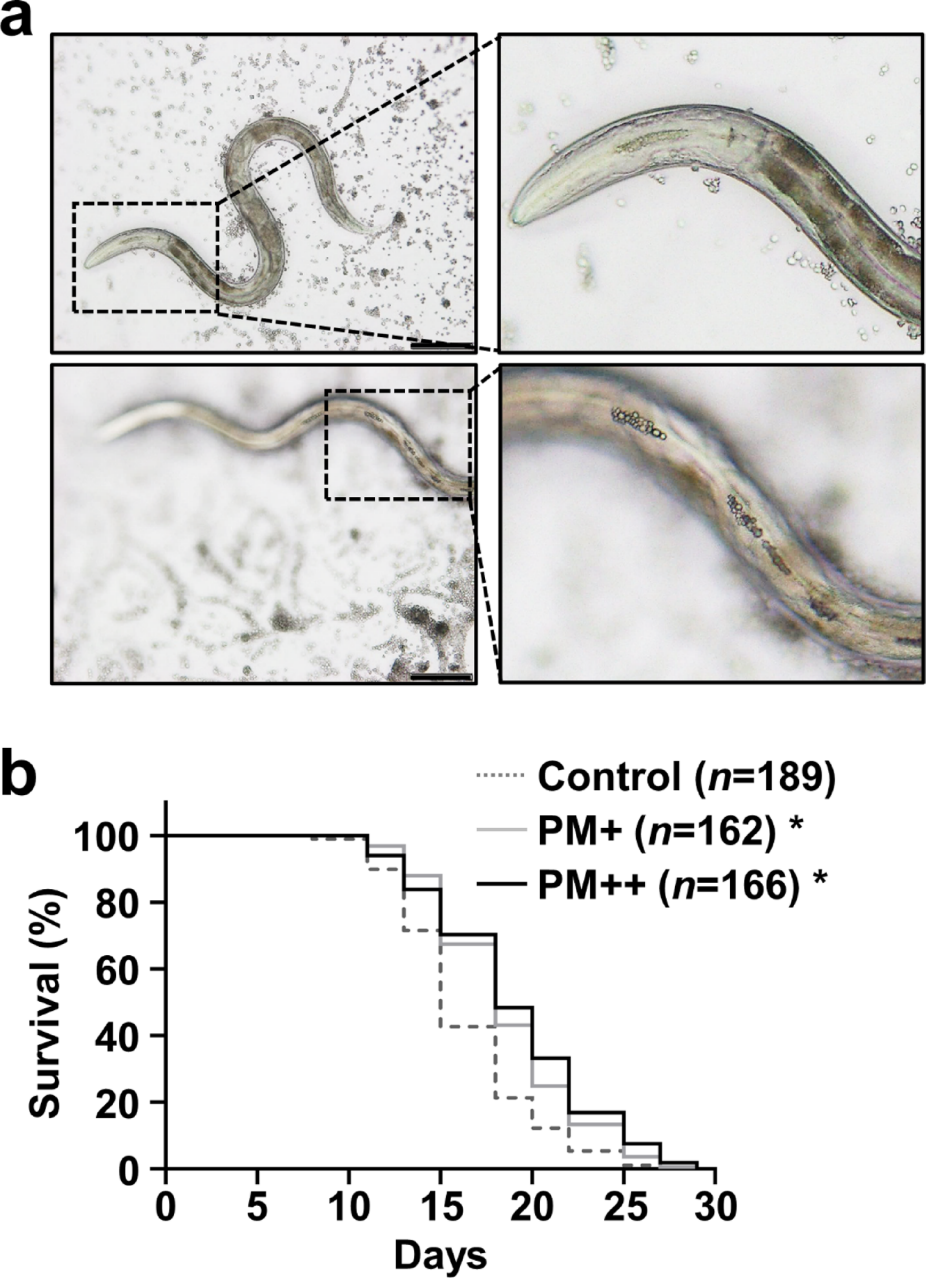
Effects of PM on the *C. elegans* lifespan. (**a**) Worms were exposed to PM suspended in S basal buffer. Their feeding behavior was observed under a light microscope (BX-50; Olympus Corp., Tokyo, Japan). Scale bars represent 100 μm (×20 magnification). **(b)** Five-day-old worms were transferred to NGM plates covered with OP50 alone or OP50 plus PM (10 or 30 mg/mL). The live and dead worms were counted three times a week. Data were evaluated using the Kaplan–Meier method. Survival differences were tested for significance using the log-rank test. Mean lifespans ± SE were the following: Control (*n* = 189), 16.3 ± 0.3 days; 10 mg/mL PM (PM+, *n* = 162), 18.6 ± 0.3 days; 30 mg/mL PM (PM++, *n* = 166), 19.0 ± 0.4 days. Significant differences compared to worms treated with OP50 alone are presented (*p < 0.05).

For initial confirmation of whether *C. elegans* ingests particulate PM, worms were exposed to PM suspended in S basal buffer [100 mM NaCl, 50 mM potassium phosphate (pH 6.0)]. During feeding, *C. elegans* pumps liquid into the intestinal lumen through rhythmic contractions of the pharynx. Observations indicated that PM, in its particulate form, was taken up gradually into the digestive tract via the pharynx (Fig. 1a). Next, to investigate PM effects on the *C. elegans* lifespan, 5-day-old adult worms were transferred to NGM plates supplemented with PM at concentrations of 10 mg/mL and 30 mg/mL, prepared as described in Materials and Methods reported herein. As shown in Fig. 1b and Supplementary Table 1, PM at concentrations of 10 mg/mL and 30 mg/mL respectively increased the mean lifespan of *C. elegans* by 14% and 16%. Until day 10, no notable difference was found among the survival rates of the three groups. However, from day 12 onward, the survival curves of the two PM-treated groups began to diverge from those of the control group fed only OP50.

**Fig. 2:**
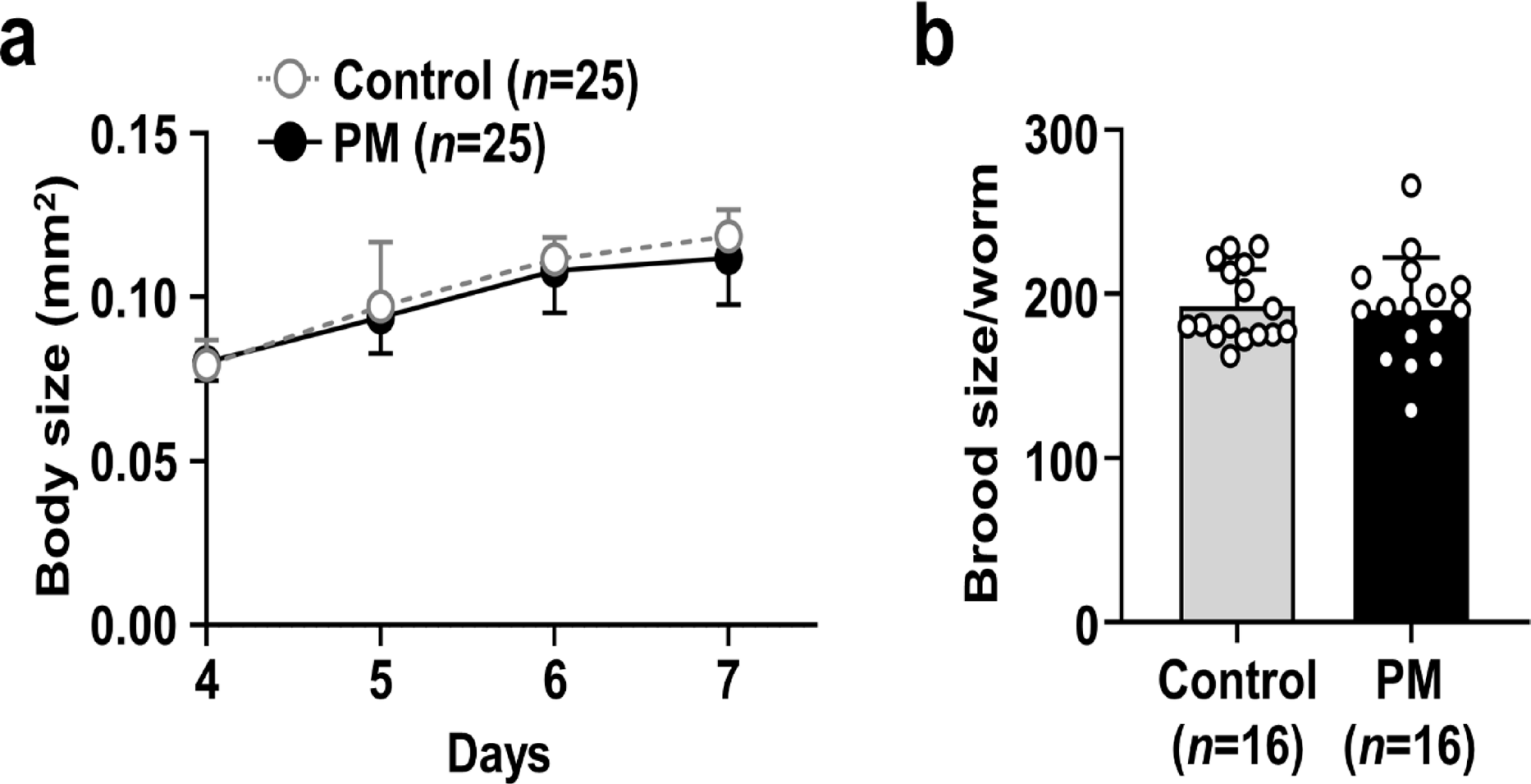
Effects of PM on body and brood sizes of *C. elegans*. Four-day-old worms were transferred to NGM plates covered with OP50 alone or OP50 plus 30 mg/mL PM. **(a)** At 4–7 days, body sizes were measured. **(b)** Worms were transferred to fresh NGM plates every 24 h. The progenies were counted after 2 days. Data represent the mean ± SE.

Caloric restriction is known to extend the lifespan of various organisms, and of *C. elegans*^28,29^. Worms subjected to caloric restriction typically exhibit reduced body size and brood size^30^. To ascertain whether the lifespan extension induced by PM was attributable to caloric restriction, the body sizes and brood sizes of PM-fed worms were compared with those of control worms fed OP50 alone. PM supplementation did not affect the worm growth curve (Fig. 2a and Supplementary Table 2). Similarly, no significant differences were found in brood size (Fig. 2b and Supplementary Table 2). Furthermore, measurement of the developmental time until the onset of egg laying showed that PM supplementation had no observable effect (Supplementary Fig. 1 and Supplementary Table 7). These findings suggest that the lifespan extension conferred by PM occurs independently of caloric restriction.

###  Effects of PM on age-related biomarkers in *C. elegans*

**Fig. 3:**
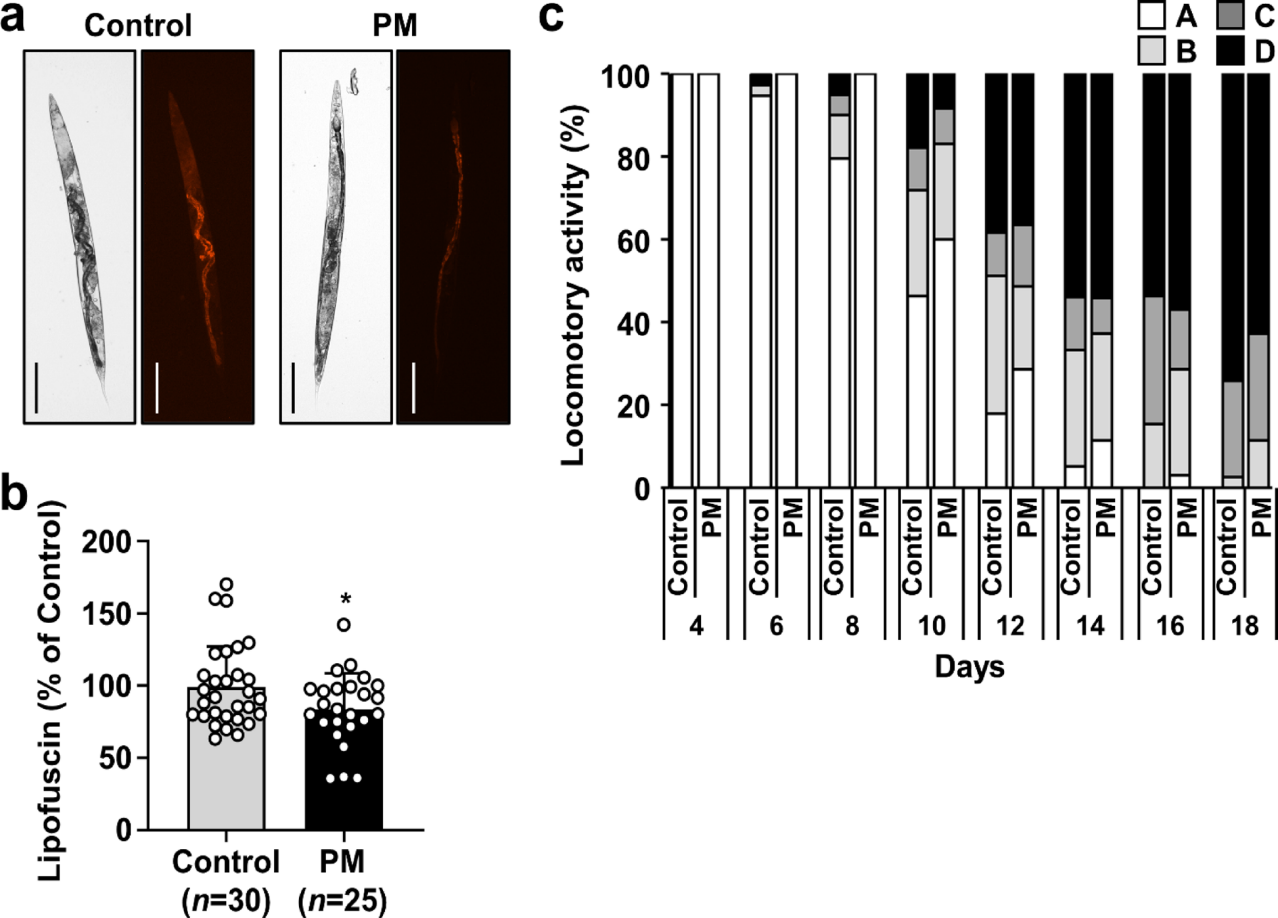
Effects of PM on lipofuscin accumulation and locomotive activity of *C. elegans*. Four-day-old worms were transferred to NGM plates covered with OP50 alone or OP50 plus 30 mg/mL PM. **(a,b)** At 14 days, lipofuscin was measured by assessing autofluorescence using an imaging system (EVOS M7000; Thermo Fisher Scientific K.K.). Representative images of each group worms are shown. Scale bars represent 100 μm (×10 magnification). Autofluorescence of lipofuscin was quantified using Image J software. Data represent the mean ± SE. Significant differences compared to worms treated with OP50 alone were determined using the Mann–Whitney *U* test (*p < 0.05). **(c)** At 4–18 days, the worms were assigned to four classes based on locomotion as described in Materials and Methods section. The locomotion was evaluated using 35–39 worms for each group.

Lipofuscin accumulation and muscle function are known to correlate with the *C. elegans* aging process^31^. Lipofuscin, a byproduct of lipid peroxidation, is commonly assessed via autofluorescence intensity. The autofluorescence intensity of worms fed with PM was significantly lower than that observed in worms fed OP50 alone (Fig. 3a and b and Supplementary Table 3). To evaluate muscle function further, locomotory scoring was performed. Locomotory scores were assessed every 48 h at the time points presented in Fig. 3c. Throughout the experiment period, the proportions of worms exhibiting vigorous movement (class A) were consistently higher in the PM-treated group than in the control group, indicating improved locomotor ability.

###  Longevity effect of PM is mediated by *clec-196*

**Fig. 4:**
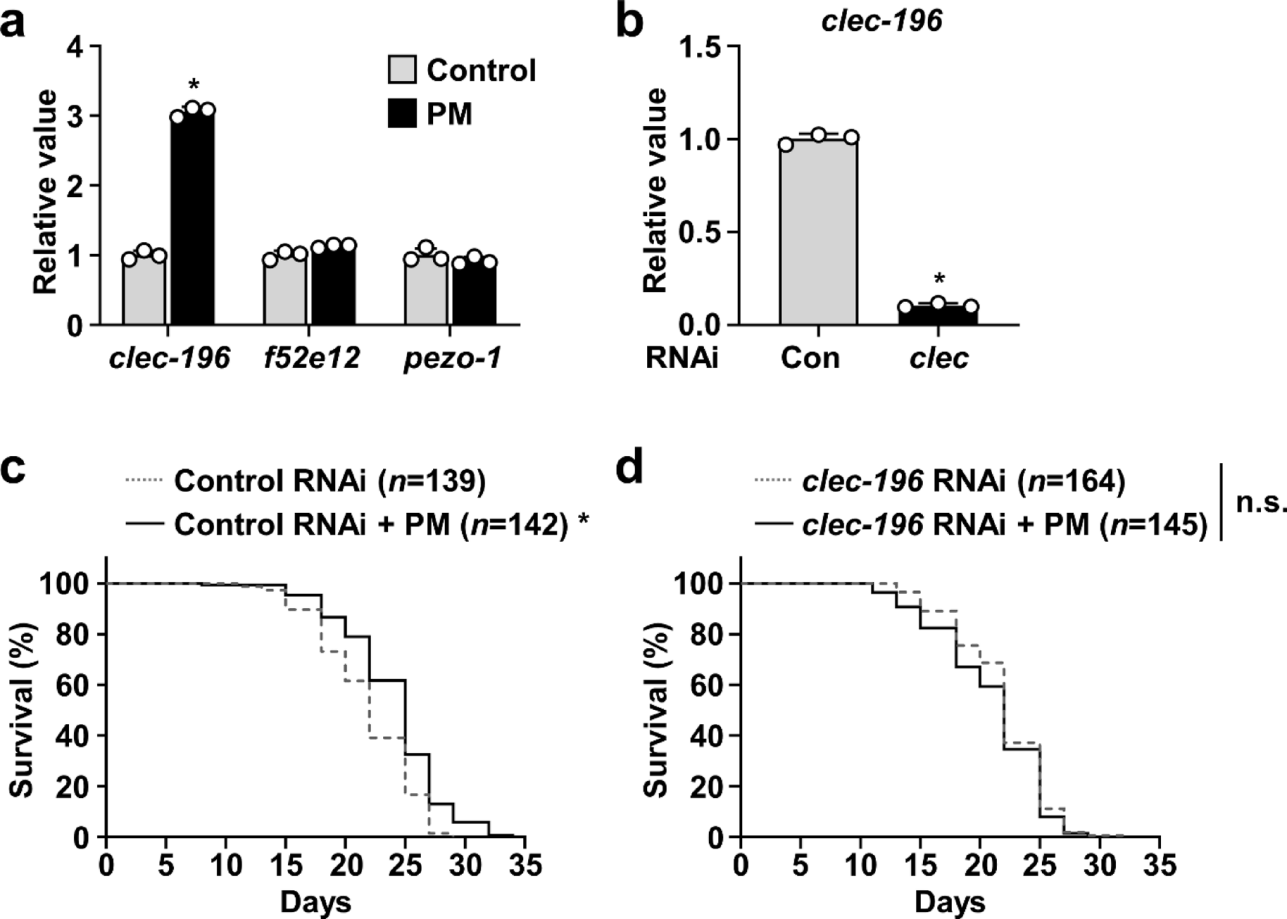
Involvement of *clec-196* in PM-mediated lifespan extension. (**a**) Four-day-old worms were transferred to NGM plates covered with OP50 alone or OP50 plus 30 mg/mL PM. After 24 h, total RNA was extracted; the expression levels of *clec-196*, *f52e12*, and *pezo-1* were evaluated using qRT-PCR. Data were normalized to the level of *act-1*. Data represent the mean ± SE (*n* = 3). Significant differences compared to worms treated with OP50 alone were determined using the Student’s *t*-test (*p < 0.05). **(b)** The expression levels of *clec-196* in RNAi-treated worms were evaluated using qRT-PCR. Data were normalized to the level of *act-1*. Data represent the mean ± SE (*n* = 3). Significant differences compared to worms treated with HT115 carrying the empty L4440 vector were determined using the Student’s *t*-test (*p < 0.05). **(c,d)** RNAi-treated worms were transferred to NGM plates covered with HT115 carrying the respective vectors alone, or with HT115 plus 30 mg/mL PM. The live and dead worms were counted three times a week. Data were calculated using the Kaplan–Meier method. Survival differences were tested for significance using the log-rank test. Mean lifespans ± SE were the following: (**c**) Control RNAi (*n* = 139), 21.8 ± 0.3 days; Control RNAi + PM (*n* = 142), 24.1 ± 0.3 days; and (** d**) *clec-196* RNAi (*n* = 164), 21.9 ± 0.3 days; *clec-196* RNAi + PM (*n* = 145), 21.0 ± 0.4 days. Significant differences compared to the corresponding HT115-treated control groups are presented (*p < 0.05).

When ingested by mice, PM granules, which are resistant to various digestive enzymes, are known to be excreted in their intact particulate form without being digested^17^. As presented in Fig. 1a, PM was also observed as particulate matter within the intestinal lumen of *C. elegans*, suggesting that it might exert its biological functions without undergoing either digestion or absorption. To elucidate the molecular mechanisms underlying PM-mediated lifespan extension, we examined the expression of *clec-196*, an ortholog of β-glucan receptor DECTIN-1, *f52e12*, an ortholog of DECTIN-2, and *pezo-1*, an ortholog of mechanosensitive ion channel PIEZO-1. Quantitative real-time PCR (qRT-PCR) analysis revealed that the expression of *clec-196* was upregulated upon PM administration (Fig. 4a and Supplementary Table 4). In contrast, no marked change was observed in the expression level of *f52e12* or of *pezo-1* following PM treatment. To investigate further whether *clec-196* is involved in PM-mediated lifespan extension, RNAi via the feeding method was conducted. As shown in Fig. 4b and Supplementary Table 4, worms fed with *Escherichia coli* HT115 (HT115) transformed with a vector expressing *clec-196* dsRNA exhibited approximately 90% suppression of *clec-196* expression compared to the control worms. Subsequently, we assessed the PM effects on the lifespans of *clec-196* knockdown worms and control worms. In control worms, similarly to wild-type animals, PM supplementation extended the lifespan considerably: a 10% increase in mean lifespan (Fig. 4c and Supplementary Table 1). In contrast, this lifespan-extending effect of PM was not found in *clec-196* knockdown worms (Fig. 4d and Supplementary Table 1). These results indicate that *clec-196* is necessary for pro-longevity effects of PM in *C. elegans*.

###  Longevity effects of PM require DAF-16

**Fig. 5:**
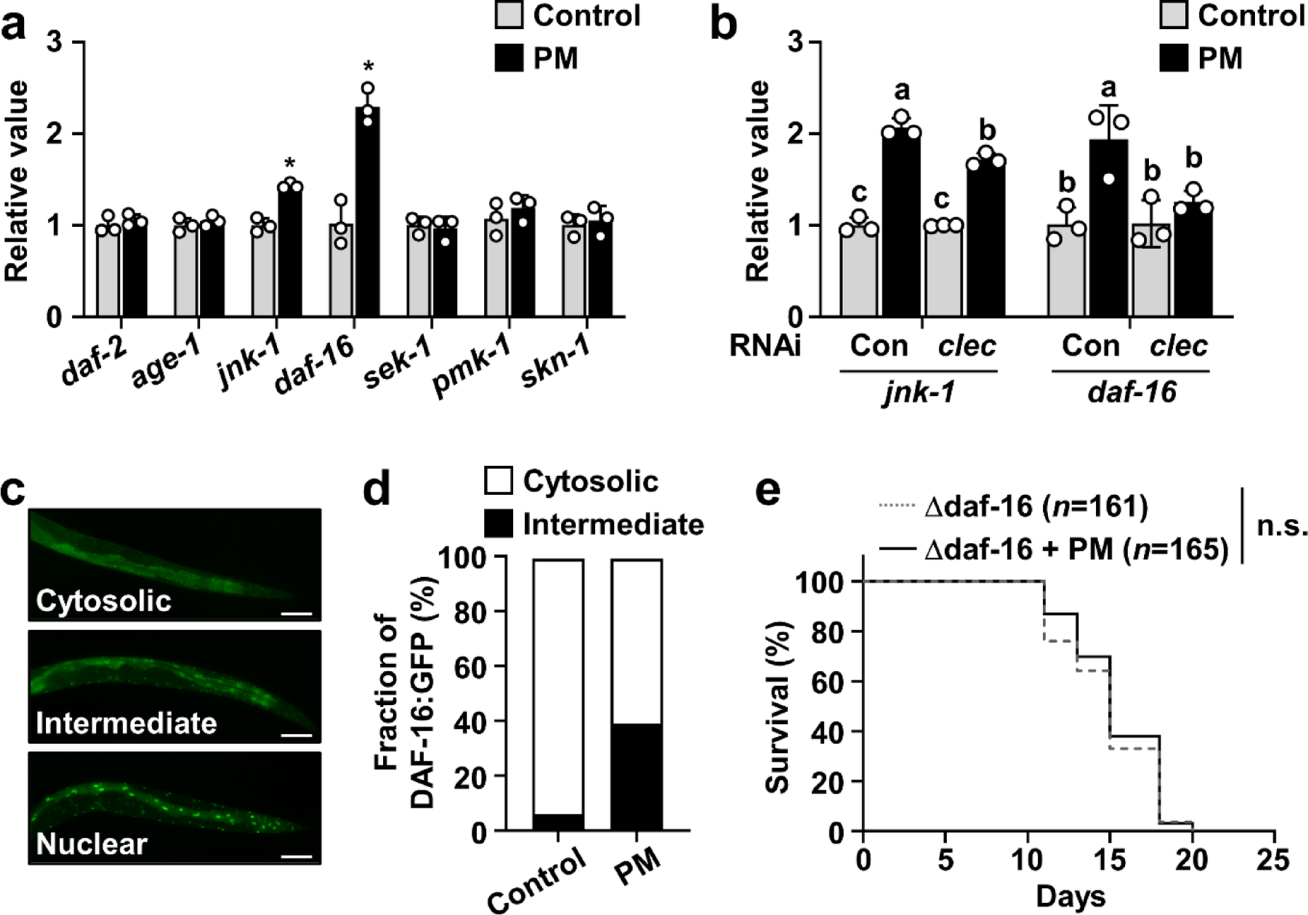
Involvement of DAF-16 in PM-mediated lifespan extension. (**a**) Four-day-old worms were transferred to NGM plates covered with OP50 alone or OP50 plus 30 mg/mL PM. After 24 h, total RNA was extracted and the expression levels of genes related to lifespan extension were evaluated using qRT-PCR. **(b)** The expression levels of *jnk-1* and *daf-16* in RNAi-treated worms were evaluated using qRT-PCR. Data were normalized to the level of *act-1*. Data represent the mean ± SE (*n* = 3). Significant differences compared to the corresponding OP50-treated control groups were determined using the Student’s *t*-test (*p < 0.05). **(c,d)** Subcellular distribution of DAF-16::GFP was detected using an imaging system (EVOS M7000; Thermo Fisher Scientific K.K.). Representative fluorescence images of TJ356 worms with cytosolic, intermediate, and nuclear localization of DAF-16::GFP are shown. Scale bars represent 50 μm (×20 magnification). Four-day-old TJ356 worms were transferred to NGM plates covered with OP50 alone or OP50 plus 30 mg/mL PM for 24 h. Bar graph showing the fraction of TJ356 worms with cytosolic or intermediate localization of DAF-16::GFP following PM treatment. **(e)** CF1038 worms were transferred to NGM plates covered with OP50 alone or OP50 plus 30 mg/mL PM from 5 days old. Kaplan–Meier survival curves are shown. Mean lifespans ± SE were the following: Δdaf-16 (*n* = 161), 14.9 ± 0.2 days; Δdaf-16 + PM (*n* = 165), 15.3 ± 0.2 days. Significant differences compared to worms treated with OP50 alone are presented (*p < 0.05).

The DAF-2/DAF-16, JNK-1/DAF-16, and p38 MAPK/SKN-1 signaling pathways are known to contribute to stress resistance, which is associated in turn with lifespan extension^32–34^. To ascertain whether these pathways are involved in the lifespan-extending effects of PM, or not, we used qRT-PCR to examine the expression of genes associated with these three pathways. As shown in Fig. 5a and Supplementary Table 5, the expression levels of *jnk-1* and *daf-16* were upregulated in response to PM treatment. It is noteworthy that these upregulations were suppressed by knockdown of *clec-196* (Fig. 5b and Supplementary Table 5). In contrast, PM supplementation had no apparent effect on the expression of *daf-2* or *age-1*, which are involved in the DAF-2/DAF-16 pathway, or on expression of *sek-1*, *pmk-1*, or *skn-1*, which are components of the p38 MAPK/SKN-1 pathway. Taken together, these results suggest that the pro-longevity effect of PM is mediated through activation of the JNK-1/DAF-16 signaling pathway via *clec-196*.

As a biological validation of the gene expression analysis results and for further evaluation of whether PM-mediated lifespan extension is associated with increased *daf-16* expression, we examined *C. elegans* strains TJ356 and CF1038. The lower panel of Fig. 5c portrays a representative image of a TJ356 worm categorized as “nuclear,” in which strong fluorescence is observed within the cell nuclei, whereas no fluorescence is detected in the soma, which indicates that the transcription factor DAF-16::GFP has translocated into the nuclei. By contrast, worms classified as “cytosolic” display weak, diffusely distributed fluorescence throughout the body (Fig. 5c, upper panel), suggesting that DAF-16::GFP remains in the cytoplasm. In the “intermediate” type, bright nuclear fluorescence is observed along with faint background fluorescence (Fig. 5c, middle panel), indicating that the transcription factor has partially translocated into the nuclei, whereas a portion remains in the cytoplasm. Multiple fluorescence images were acquired from TJ356 worms treated with PM. Each worm was categorized according to the classification criteria presented above. Based on these observations, the nuclear translocation rate of DAF-16 was calculated. Although no worm in either the control or PM-treated group was classified as “nuclear,” the proportions of worms categorized as “intermediate” were markedly different between the two groups: 6.5% in control worms and 39.1% in PM-treated worms (Fig. 5d). For further confirmation of the involvement of DAF-16 in PM-mediated lifespan extension, we assessed the effect of PM treatment on the lifespan of the DAF-16 mutant strain CF1038. The results demonstrated that the lifespan of CF1038 worms fed PM was comparable to that of control CF1038 worms fed OP50 alone (Fig. 5e and Supplementary Table 1). These findings suggest that the lifespan-extending effect of PM is dependent on the DAF-16 signaling pathway.

### PM enhances DAF-16-mediated antioxidant pathway

**Fig. 6:**
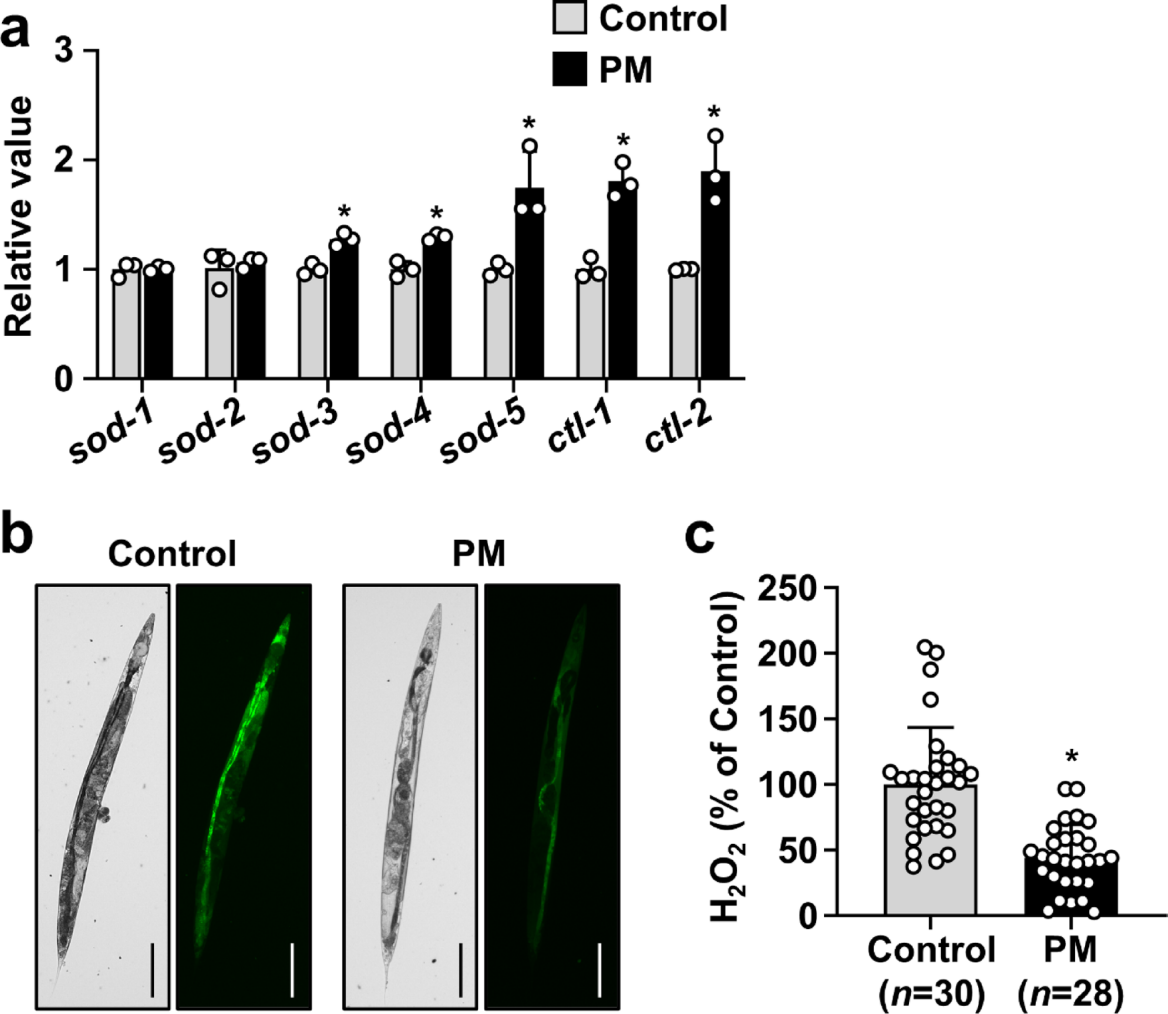
Involvement of antioxidant pathway in PM-mediated lifespan extension. (**a**) Four-day-old worms were transferred to NGM plates covered with OP50 alone or OP50 plus 30 mg/mL PM. After 24 h, total RNA was extracted. The expression levels of genes related to antioxidant pathway were evaluated using qRT-PCR. Data represent the mean ± SE. Significant differences compared to worms treated with OP50 alone were determined using the Student’s *t*-test (*p < 0.05). **(b,c)** At 14 days old, accumulation of H_2_O_2_ was detected using the fluorescent probe BES-H_2_O_2_-Ac with an imaging system (EVOS M7000; Thermo Fisher Scientific K.K.). Representative images of worms fed each bacterial strain are shown. Scale bars represent 100 μm (×10 magnification). Fluorescence intensities of H_2_O_2_ was quantified using Image J software. Data represent the mean ± SE. Significant differences compared to worms treated with OP50 alone were determined using the Mann–Whitney *U* test (*p < 0.05).

To investigate further whether the DAF-16 signaling pathway is involved in PM-mediated lifespan extension, or not, we measured the expression levels of antioxidant-related genes downstream of DAF-16. As Fig. 6a and Supplementary Table 6 show, the expression levels of *sod-3*, *sod-4*, *sod-5*, *ctl-1*, and *ctl-2* were elevated in worms fed PM compared to those fed OP50 alone. In contrast, the expression of *sod-1* and *sod-2* was unaffected by PM treatment. Subsequently, worms fed PM were subjected to staining with BES-H_2_O_2_-Ac, a fluorescent probe for hydrogen peroxide (H_2_O_2_) detection. The intestinal tract and surrounding tissues exhibited a marked degree of fluorescence in control worms fed OP50 alone, whereas this fluorescence was much lower in worms fed PM (Fig. 6b and c and Supplementary Table 6). These findings suggest that the lifespan-extending effect of PM is associated with activation of an antioxidant pathway mediated by DAF-16.

## Discussion

During the *C. elegans* aging process, age-related biomarkers such as lipofuscin accumulation and locomotory activity undergo characteristic changes^31^. Results of the present study demonstrated that PM supplementation extended the lifespan of *C. elegans*, reduced lipofuscin accumulation, and maintained vigorous locomotion. Furthermore, the pro-longevity effect of PM was suggested to be mediated through activation of an antioxidant pathway via DAF-16, initiated by *clec-196*, an ortholog of β-glucan receptor DECTIN-1.

Caloric restriction is recognized as an effective intervention for lifespan extension not only in numerous non-mammalian species but also in mammals, including primates^29,30^. Results of the present study demonstrated that PM supplementation had no effect on the growth curve or brood size of *C. elegans*, suggesting that caloric restriction is not involved in PM-mediated lifespan extension. *Escherichia coli*, which is commonly used as a standard diet for *C. elegans*, is a rod-shaped bacterium with a length of approximately 2–4 μm. Similarly, lactic acid bacteria, which are also used frequently in feeding assays involving *C. elegans*, are generally rod-shaped or coccoid-shaped, and of micrometer-range size. Moreover, a study by Shang et al. comparing the ingestion of microplastic particles of 1 μm and 5 μm diameter found no significant difference in intake between the two particle sizes^35^. Taken together, these findings suggest that the presence of PM does not affect food intake in *C. elegans*. Moreover, for this study, we directly observed the PM particle ingestion by the worms.

Ishibashi et al. reported that PM binds directly to the β-glucan receptor DECTIN-1 in its particulate form, as demonstrated by in vitro assays using both human and murine DECTIN-1^13^. In *C. elegans*, *clec-196* has been identified as an ortholog of the β-glucan receptor DECTIN-1^36^. The present study demonstrated that PM supplementation upregulates the expression of *clec-196*, and that RNAi-mediated knockdown of *clec-196* eradicates the lifespan-extending effect of PM. These findings suggest that *clec-196*, similarly to DECTIN-1, might function as a receptor for PM. However, no report has described a study elucidating the structural or functional characteristics of *clec-196*. DECTIN-1 binds β-glucans via a CTLD, which corresponds to amino acids 120–243 in the human protein^37^. In *clec-196*, the region corresponding to the CTLD spans amino acids 29–143. Although the sequence identity within this domain is low (24%) compared to that of human DECTIN-1, all six cysteine residues characteristic of CTLDs are conserved. Furthermore, among the three amino acid residues in human DECTIN-1 critical for ligand binding (Trp222, His224, and Tyr229)^38^, Tyr229 is conserved in *clec-196*, and His224 is substituted with Tyr, an amino acid with similar properties. Additionally, Packer et al. reported that *clec-196* is expressed predominantly in the intestinal cells of *C. elegans*^39^. An earlier report by Townes et al.. is particularly interesting: They employed various machine learning algorithms to identify genes associated with lifespan extension. Their findings indicate that *clec-196* was ranked among the top ten candidate genes, even though its physiological functions remain largely uncharacterized^40^. Collectively, these observations suggest strongly that PM exerts its physiological effect of lifespan extension through recognition by *clec-196* in intestinal cells.

Several evolutionarily conserved signaling pathways regulate the *C. elegans* lifespan. Among these, one central transcriptional regulator is DAF-16, an ortholog of the forkhead box O (FoxO) subfamily of transcription factors, which governs the expression of numerous genes associated with longevity^25,41^. DAF-16 is negatively regulated by the IIS pathway, which is initiated by the binding of insulin-like peptides to the receptor DAF-2 and transduced via AGE-1, the *C. elegans* ortholog of phosphatidylinositol 3-kinase^42^. In contrast, it is positively regulated by JNK-1, the *C. elegans* ortholog of c-Jun N-terminal kinase (JNK), a member of the MAPK family^33^. Moreover, qRT-PCR analysis revealed that PM supplementation did not affect the IIS pathway, but rather activated the JNK pathway, resulting in increased expression of *daf-16*. Furthermore, analysis of subcellular localization using the DAF-16::GFP transgenic strain TJ356 and lifespan assays using the DAF-16 loss-of-function mutant CF1038 demonstrated that DAF-16 is necessary for the lifespan-extending effects of PM. In addition, the PM-induced upregulations of *daf-16* and *jnk-1* expression were eradicated by RNAi-mediated knockdown of *clec-196*. It has also been reported that the JNK signaling pathway functions downstream of DECTIN-1 in mammals^43^. However, while *daf-16* induction by PM was abolished by *clec-196* knockdown, the effect on *jnk-1* expression was limited, suggesting that other factors may also contribute to the regulation of *daf-16* downstream of PM and *clec-196*. Nevertheless, these findings collectively identify a novel signaling axis in which the JNK-1/DAF-16 pathway operates downstream of *clec-196* in *C. elegans*.

Activation of DAF-16, a transcription factor involved in various physiological processes, including antioxidant pathways, has been shown to promote lifespan extension^25,41^. In fact, in *C. elegans* fed with PM, the expression of several antioxidant-related genes was upregulated. The accumulation of H_2_O_2_ was suppressed. Earlier reports have described that the expression of *sod-3*, *ctl-1*, and *ctl-2* is positively regulated by DAF-16, whereas *sod-1* and *sod-2* are negatively regulated by DAF-16. Additionally, *sod-4* and *sod-5* have been proposed as putative target genes of DAF-16^44^. No marked change was observed in the expression of either *sod-1* or *sod-2* in this study, but accumulated evidence suggests that the antioxidant pathway mediated by DAF-16 contributes to the lifespan-extending effects of PM.

In this study, we demonstrated that PM extends lifespan in *C. elegans* through recognition by *clec-196* and subsequent activation of a DAF-16–mediated antioxidant signaling pathway. These results support the recently reported anti-aging effects of *E. gracilis* with enhanced PM production generated by the CRISPR/Cas9 system and provide new mechanistic insights into the lifespan-extending effects of PM^45^. Importantly, this mechanism is evolutionarily conserved, suggesting its potential relevance to higher organisms. Indeed, activation of antioxidant pathways in response to PM intake has also been observed in humans^18^, mice^17^, and rats^19^, further supporting the applicability of our findings beyond the nematode model. It is also well established that indigestible carbohydrates exert various health-promoting effects through the modulation of gut microbiota. However, because of the wide inter-individual variation in gut microbial composition, individual responses to such dietary components can vary considerably, resulting in the existence of both responders and non-responders. Given that *C. elegans* is exposed only to *E. coli*, the demonstration of such functionality in this organism provides a valuable example indicating that PM might exert its health benefits independently of the gut microbiota. These findings suggest that PM holds potential as a novel functional food ingredient with microbiota-independent efficacy. It is particularly suitable for populations with diverse gut microbial profiles.

## Materials and methods

### *C. elegans* strains and maintenance

The wild-type *C. elegans* strain Bristol N2 and its derivative mutant strains were obtained from the Caenorhabditis Genetics Center (CGC; MN, USA). The mutants used for this study were NL2099 *rrf-3 (pk1426) II*, CF1038 *daf-16 (mu86) I*, and TJ356 *zls356(DAF-16::GFP + rol-6)*. Worms were maintained using standard techniques on 9 cm nematode growth medium (NGM) plates seeded with OP50 an incubator at 20 °C^46^. NGM plates contained 2.0% (w/v) peptone, 1.8% (w/v) agar, 25 mM potassium phosphate, 1.0 mM MgSO_4_, 1.0 mM CaCl_2_, and 5.0 µg/mL cholesterol. OP50 was grown in Luria–Bertani (L.B.) broth overnight at 37 °C. Eggs were recovered from young adult worms by exposure to a lysis solution (0.6% sodium hypochlorite, 200 mM sodium hydroxide). The egg suspension was incubated 12–14 h at 20 °C to facilitate hatching and growth on NGM plates seeded with OP50 for 3 days. Some of the experiments were conducted with the approval of the Institutional Committee for Biosafety of Recombinant DNA Experiments at Ishikawa Prefectural University (Approval No. 24 − 13).

###  Administration of paramylon

For this study, PM derived from the *E. gracilis* EOD-1 (Kobelco Eco-Solutions Co., Ltd., Kobe, Japan) was used. *E. gracilis* EOD-1, a high-PM-accumulating strain, stores 70–80% PM within its biomass. Given that PM from the *E. gracilis* EOD-1 exists as granules of approximately 3 μm diameter^47^, its administration to *C. elegans* was performed in accordance with protocols established in earlier studies involving microplastic particle exposure^35^. Briefly, 4-day-old or 5-day-old young adult worms were transferred onto NGM plates coated with a PM suspension that had been prepared by dispersing PM in an OP50 culture to achieve final concentration of 10 or 30 mg/mL. These concentrations were determined based on our earlier studies in which indigestible oligosaccharides were administered to *C. elegans*^48^. In addition, regarding the upper concentration limit, we found that observation with a stereomicroscope was technically difficult at concentrations above 50 mg/mL; therefore, we referred to the study by Gao et al. using oat powder containing b-glucan and set the upper concentration at 30 mg/mL^49^.

###  Determination of the *C. elegans* lifespan

Four-day-old young adult worms were treated using 5-fluorodeoxyuridine (0.5 mg/mL) to prevent progeny production^50^. The resultant synchronized hermaphrodites were transferred to NGM plates (10 worms per 35 mm plate) covered with OP50 alone or OP50 plus PM (10 or 30 mg/mL). The plates were incubated at 20 °C. Then live and dead worms were counted three times a week. Worms were considered “dead” when they failed to respond to a gentle touch with a worm picker. Worms that crawled off the plate or showed non-natural death, such as internal hatching or adhering to the wall of the plate, were considered “lost.” Experiments were performed at least in triplicate, and the “lost” individuals were excluded from the analysis of lifespan.

###  Measurements of body and brood sizes

Four-day-old young adult worms were placed on NGM plates (one worm per 35 mm plate) covered with OP50 alone or OP50 plus 30 mg/mL PM. The live worm body sizes were measured every 24 h until 7 days of age. Then they were analyzed as reported earlier^44^. For brood size measurement, the parental worms were transferred every 24 h to fresh NGM plates until the end of the reproductive period. After the resulting progenies were left to develop for 2 days, they were counted.

###  Measurement of developmental time

The time from egg to the first egg-lay was defined as the developmental time of the worm. Developmental time was measured using the previously described method with minor modifications^51^. Four-day-old young adult worms were placed on NGM plates seeded with OP50 and allowed to lay eggs for 30 min. Individual eggs were then transferred to separate NGM plates seeded with OP50 alone or OP50 plus 30 mg/mL PM. Beginning at 67 h after egg laying, worms were examined every hour to determine the onset of egg laying, which was recorded as the developmental time.

### Lipofuscin accumulation

The autofluorescence of intestinal lipofuscin was measured as an index of senescence. Four-day-old young adult worms were placed on NGM plates covered with OP50 alone or OP50 plus 30 mg/mL PM until they became 14-day-old adult worms. After randomly selected worms were washed three times with S basal buffer, they were anesthetized with 100 mM sodium azide. Lipofuscin autofluorescence images were detected with excitation at 542 nm and emission at 593 nm using a microscope (EVOS M7000; Thermo Fisher Scientific Inc., MA, USA). To calculate the lipofuscin-positive area, densitometry measurements were taken using ImageJ software.

###  Locomotory scoring

Four-day-old young adult worms were placed on NGM plates (one worm per 35 mm plate) covered with OP50 alone or OP50 plus 30 mg/mL PM. Locomotory assay of worms was performed every 48 h until 18 days of age using a scoring method described in an earlier report^52^. Worms were classified according to a four-point scale: class “A” worms showed spontaneous movement or vigorous locomotion in response to prodding; class “B” worms did not move unless prodded or appeared to have uncoordinated movement; class “C” worms moved only their head or tail in response to prodding; and class “D” worms were dead.

### Quantitative real-time PCR

Four-day-old young adult worms were placed on NGM plates covered with OP50 alone or OP50 plus 30 mg/mL PM for 24 h. Worms were collected and washed three times with sterilized S basal buffer. Then, total RNA was extracted and reverse-transcribed. The resultant cDNA was subjected to qRT-PCR using each specific primer described in Table 1. PCR was performed using a PowerUP SYBR Green PCR Master Mix and a real-time PCR system (StepOnePlus; Applied Biosystems Inc., CA, USA). PCR conditions were denatured as 95 °C for 15 s, with primer-annealing and elongation at 60 °C for 1 min, with subsequent melting curve analysis for which the temperature was increased from 60 °C to 95 °C. The Ct values were transformed into relative quantification data using the 2^− ΔΔCt^ method. Data were normalized to the *act-1* endogenous control.

**Table 1:** Primer used for qRT-PCR assays.

Gene	Forward	Reverse
*act-1*	5’-CACGGTATCGTCACCAACTG-3’	5’-GCTTCAGTGAGGAGGACTGG-3’
*clec-196*	5’-ATTCGAGGCCGTCAGGAAT-3’	5’-TTCGGCTTGCATCCAAGTG-3’
*f52e12*	5’-TTGACGAGACTAAGGAAACATGGA-3’	5’-CCACTGCCATGCACCACTT-3’
*pezo-1*	5’-TCCACGTGGTGGTCCACAT-3’	5’-AGCGCCGAGTAGAATATGAGAAA-3’
*daf-2*	5’-GCCCGAATGTTGTGAAAACT-3’	5’-CCAGTGCTTCTGAATCGTCA-3’
*age-1*	5’-GGGACCTTGACGCGAATCT-3’	5’-CGGCCGGGAAATCGA-3’
*jnk-1*	5’-TCGTATCCGTCACATCCAGGTA-3’	5’-AGGATGCGAGCAGAGTGTTGT-3’
*daf-16*	5’-TCCTCATTCACTCCCGATTC-3’	5’-CCGGTATATTCATGAACGTG-3’
*sek-1*	5’-TGGCAAACACATTCCAGAGC-3’	5’-AGTCTTGGCCATGCTGTTTG-3’
*pmk-1*	5’-ACTCGCCGTGATTTCAAACG-3’	5’-CAGTTGGACGACGATCTGGA-3’
*skn-1*	5’-TCAGGACGTCAACAGCAGAC-3’	5’-CGTGGAGATTCCGAAGAGAG-3’
*sod-1*	5’-CTGCCTGCGGTGTCATTG-3’	5’-GAGACGCGATTCAGGTAGTCACT-3’
*sod-2*	5’-AGGATCCACTTGAGGCAACAA-3’	5’-TGCTCCCAGACGTCAATTCC-3’
*sod-3*	5’-CCTGTGCAAACCAGGATCCT-3’	5’-CCCAAACGTCAATTCCAAAAA-3’
*sod-4*	5’-TTGGGACGCGGTACTTCAG-3’	5’-GCAAGTCGGCTTCCAGCAT-3’
*sod-5*	5’-GCCTCTTCGGAGCGAACA-3’	5’-TCTCGATCGACGTGGACAAC-3’
*ctl-1*	5’-GCCGGAGCCCATGGAT-3’	5’-CGGCCTTACAGTACTTGGTGATG-3’
*ctl-2*	5’-GGTCACCCATGACATCTCCAA-3’	5’-TGCTTCCCGACCTTGTTGA-3’

###  RNA interference experiments

RNA interference was conducted based on a method reported earlier, with minor modifications^53^. Briefly, HT115 harboring either the L4440 vector expressing *clec-196* dsRNA (Clone: sjj_F26D10.12; DNAFORM Inc., Yokohama, Japan) or the empty L4440 vector (#1654; Addgene Inc., MA, USA) as a negative control was cultured overnight in L.B. broth. Subsequently, 1.0 mL of the bacterial suspension was spread onto NGM plates supplemented with 100 µg/mL ampicillin and 1.0 mM isopropyl-β-D-thiogalactopyranoside. The plates were left at room temperature overnight. Synchronized L1 larvae of the RNAi-sensitive *C. elegans* strain NL2099 were then transferred to the prepared NGM plates and were cultivated until 4-day-old young adult worms. The worms were subsequently used for the respective assays. To assess the knockdown efficiency, total RNA was extracted from 4-day-old young adult worms and was analyzed using qRT-PCR.

###  Subcellular distribution of DAF-16

Four-day-old young adult worms of TJ356 strain were placed on NGM plates covered with OP50 alone or OP50 plus 30 mg/mL PM for 24 h. Randomly selected worms were washed three times with S basal buffer and were anesthetized with 100 mM sodium azide. Changes in GFP fluorescence in DAF-16::GFP worms were observed with excitation at 470 nm and emission at 510 nm using the imaging system (EVOS M7000; Thermo Fisher Scientific K.K.). GFP expression patterns were classified into three categories: cytosolic, intermediate, and nuclear, as described in an earlier report^54^. The distribution ratio of each GFP expression pattern was calculated for each treatment group. As a positive control for the nuclear pattern, 5-day-old worms subjected to heat stress at 35 °C for 2 h were used.

### Fluorescence staining of H_2_O_2_

To detect cellular H_2_O_2_ levels in worms, fluorescent probe BES-H_2_O_2_-Ac (Fujifilm Wako Pure Chemical Corp., Tokyo, Japan) was used. For use as a staining solution, the BES-H_2_O_2_-Ac was diluted to a final concentration of 200 µM using S basal buffer. Four-day-old young adult worms were placed on NGM plates covered with OP50 alone or OP50 plus 30 mg/mL PM until they developed to 14-day-old adult worms. After randomly selected worms were washed three times with S basal buffer, they were treated with 450 µL of staining solution for 1 h. After washing with S basal buffer, the worms were anesthetized using 100 mM sodium azide. The H_2_O_2_ levels were observed with excitation at 470 nm and emission at 510 nm using an imaging system (EVOS M7000; Thermo Fisher Scientific K.K.).

### Statistical analysis

After *C. elegans* survival was calculated using the Kaplan–Meier method, survival differences were tested for significance using the log-rank test. For comparisons between the Control and PM groups, normality was first assessed using the Shapiro–Wilk test. When the data did not follow a normal distribution, significance was determined using the Mann–Whitney *U* test; otherwise, Student’s *t*-test was applied. Comparisons among four groups were performed using one-way analysis of variance (ANOVA) with Tukey’s post hoc testing. Statistical analyses were performed using software (GraphPad Prism ver. 8.0.1; GraphPad Software Inc., CA, USA). All results are expressed as mean ± SE. Differences for which *p* < 0.05 were inferred as significant.

## Supplementary Information

Below is the link to the electronic supplementary material.

Supplementary Material 1

Supplementary Material 2

Supplementary Material 3

Supplementary Material 4

Supplementary Material 5

Supplementary Material 6

Supplementary Material 7

Supplementary Material 8

Supplementary Material 9

## Data Availability

All data generated or analyzed during this study are included in this published article (and its supplementary information files). It can also contact the corresponding author for additional requests.
