# Supplementary material for: Paramylon isolated from Euglena gracilis EOD-1 extends lifespan through activation of DAF-16-mediated antioxidant pathway via clec-196 in Caenorhabditis elegans
Source: Sci Rep. 2025 Nov 26;15:42202. doi: 10.1038/s41598-025-26199-3 (PMC12657932; doi:10.1038/s41598-025-26199-3)
Supplement: Supplementary file 1 — Supplementary Material 1 [file 41598_2025_26199_MOESM1_ESM.doc]

**Supplementary information**

**Figure legends**

**Supplementary Figure 1. Effect of PM on developmental time of *C. elegans*.** Four-day-old worms were transferred to NGM plates covered with OP50 and allowed to lay eggs for 30 min. Individual eggs were then transferred to separate NGM plates seeded with OP50 alone or OP50 plus 30 mg/mL PM. Beginning at 67 h after egg laying, worms were examined every hour to determine the onset of egg laying, which was recorded as the developmental time. Data represent the mean ± SE.
