# Supplementary figures and images for: Paramylon isolated from Euglena gracilis EOD-1 extends lifespan through activation of DAF-16-mediated antioxidant pathway via clec-196 in Caenorhabditis elegans
Source: Sci Rep. 2025 Nov 26;15:42202. doi: 10.1038/s41598-025-26199-3 (PMC12657932; doi:10.1038/s41598-025-26199-3)

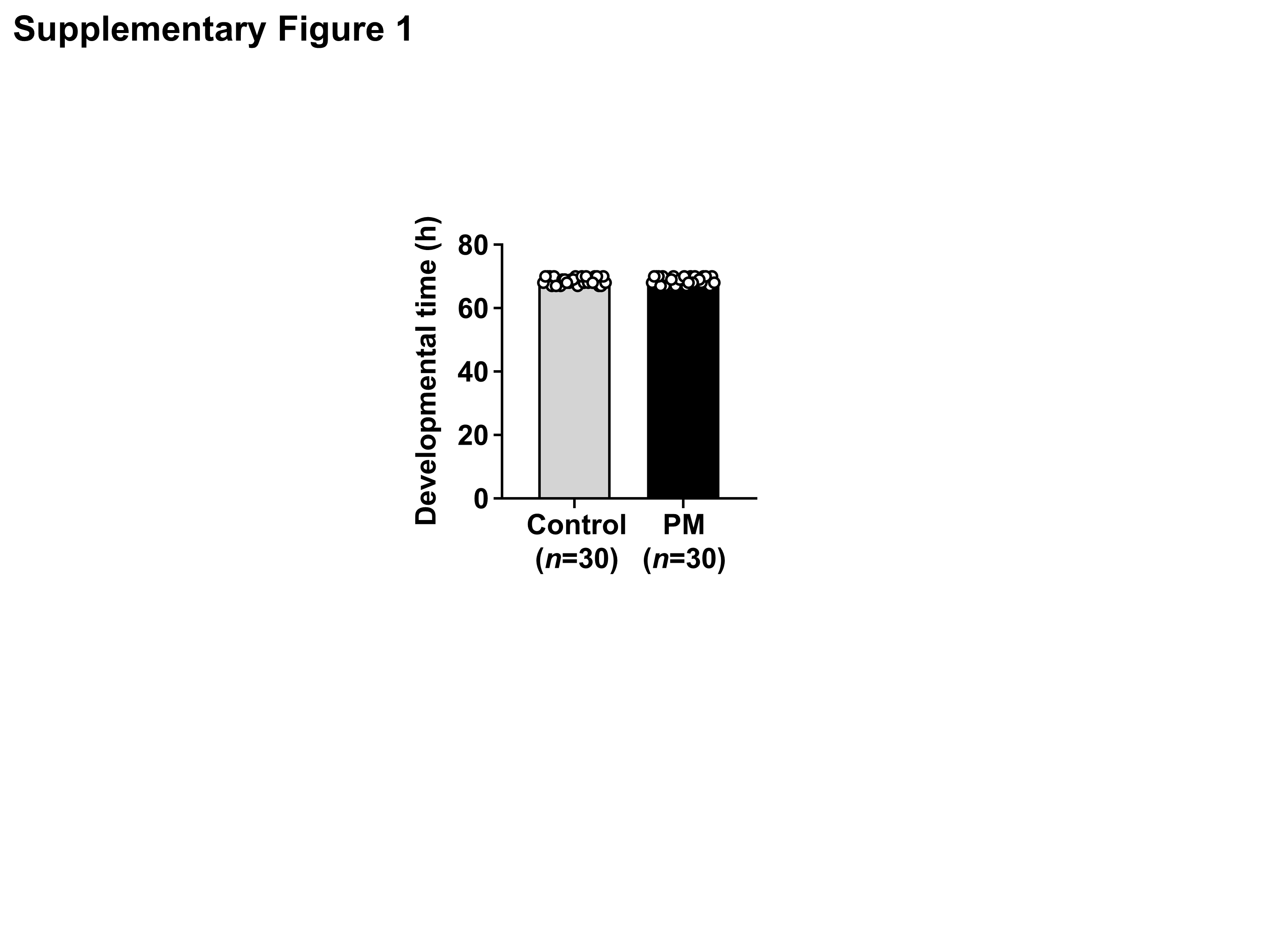

Supplement: Supplementary file 2 — Supplementary Material 2 [file 41598_2025_26199_MOESM2_ESM.tif]
